# Folic Acid Receptor-Mediated Targeting Enhances the Cytotoxicity, Efficacy, and Selectivity of *Withania somnifera* Leaf Extract: *In vitro* and *in vivo* Evidence

**DOI:** 10.3389/fonc.2019.00602

**Published:** 2019-07-04

**Authors:** Yue Yu, Jia Wang, Sunil C. Kaul, Renu Wadhwa, Eijiro Miyako

**Affiliations:** ^1^Department of Materials and Chemistry, Nanomaterials Research Institute, National Institute of Advanced Industrial Science and Technology, Tsukuba, Japan; ^2^DAILAB, DBT-AIST International Center for Translational and Environmental Research (DAICENTER), AIST, Tsukuba, Japan; ^3^Graduate School of Life and Environmental Sciences, University of Tsukuba, Ibaraki, Japan

**Keywords:** *Withania somnifera*, folic acid, i-Extract, nanomedicine, chemotherapy

## Abstract

Nanomedicine holds great potential for drug delivery to achieve more effective and safer cancer treatment. Earlier, we reported that the alcoholic extract of *Withania somnifera* leaves (i-Extract) has selective cancer cell killing activity. Herein, we developed a folate receptor-targeting i-Extract nanocomplex (FRi-ExNC) that suspends well in water and possesses enhanced selective anticancer activity in both *in vitro* and *in vivo* assays. Comparative analyses of folate receptor (FR)-positive and -negative cells revealed that FRi-ExNC caused a stronger decrease in Cyclin D/Cdk4 and anti-apoptotic protein Bcl-2, as well as a higher increase in the growth arrest regulating protein p21^WAF1^ and pro-apoptotic protein PARP-1, in FR-enriched cancer cells. Our results demonstrate that FRi-ExNC could be a natural source-based nanomedicine for targeted cancer therapy.

## Introduction

*Withania somnifera* is a popular herb (also called Indian ginseng) used in Indian traditional Ayurvedic medicine and health tonics. Although it is known for a variety of health-promoting effects and possesses high therapeutic potential, its mechanisms of action are only beginning to be addressed in laboratories. We reported earlier that the i-Extract possesses potent anti-tumor activity that was assigned to its active components, withanolides. Among them, Withaferin-A and Withanone were found to possess anticancer activity; whereas i-Extract and Withanone have been shown to be selectively cytotoxic to cancer cells, Withaferin-A causes toxicity to normal cells as well (1–5). Both bioinformatics and wet lab studies have demonstrated that both Withaferin-A and Withanone cause death of cancer cells through multi-target mechanisms (6–11). Interestingly, the water extract of *Withania somnifera* leaves was also shown to possess anticancer activity that functions through bioactives and mechanisms that are different to that of the i-Extract (12, 13). Of note, we found that Withaferin-A has the capability to kill telomerase-negative ALT (Alternative Lengthening of Telomeres) cancer cells (14) suggesting that a formulation containing Withaferin-A will be useful for the treatment of complex and aggressive cancers. With this view, a combination of Withanone and Withaferin-A in a 20:1 ratio was generated. This combination of Withanone and Withaferin-A was demonstrated to retain selective toxicity to cancer cells and anti-metastasis potential (15). Most recently, we found that although the methoxy derivative of Withaferin-A lacks anticancer activity, it protects normal cells against a variety of stresses (16). In view of the above information, i-Extract containing a variety of bioactives is considered to be more potent than the individual compounds and warrants further studies on its composition and delivery.

Clinical use of *Withania somnifera* has not been approved as of yet, due to the difficulty in identifying the real active formula in the mixture. In addition, insufficient targeting effects and the poor water solubility of i-Extract are also challenging issues. Nanomedicine offers a highly potential merger of medicine and nanotechnology for diagnostic as well as therapeutic purposes (17). Through targeted and efficient delivery, the engineered molecules and their derivatives have been seen to offer better therapeutic potential. For example, use of polyethylene glycol (PEG)-coated polymer nanocarriers (18–22) has been shown to enhance the action of drugs. Furthermore, the nanocarriers can effectively encapsulate various types of small molecules with different physicochemical features including hydrophilicity (23), hydrophobicity (24), electrical charge (25), and size (26). Enhanced permeability and a retention (EPR) effect has also been reported and serves as a key rationale for using nanocarriers to treat solid tumors (27–29). However, the targeted and uniform delivery of the nanocarriers to tumors in sufficient quantities requires optimization. An ideal nanocarrier should be equipped with selective ligands that are highly or exclusively expressed on target tumor cells, and thus endow the carriers with specific targeting capabilities.

Folate receptor alpha (FR-α) is a glycosylphosphatidylinositol cell surface-anchored glycoprotein, overexpressed in 90% ovarian carcinomas and many types of epithelial cancers (30–33). It binds to folic acid, mediates its intracellular transport via receptor-mediated endocytosis, and is essential for proliferation of these cancer cell types (34, 35). On the other hand, normal cells and tissues have limited FR-α expression that is restricted largely to the apical surface of the epithelial tissue, where it is inaccessible to the circulating drugs (36–39). Due to such limited expression in normal tissues, FR-α has been considered as a candidate for targeted delivery of anticancer drugs (40–42).

In the present study, we developed a folate receptor-targeting i-Extract nanocomplex that possesses enhanced selective cancer cell killing activity. We provide evidence that in contrast to i-Ex, FRi-ExNC is water soluble and shows a 3-fold higher cellular uptake by FR-enriched cancer cells *in vitro*. *In vivo* tumor assays also supported stronger tumor suppression capability of FRi-ExNC suggesting that may serve as an efficient and safe natural drug candidate for cancer treatment.

## Materials and Methods

### Preparation of FRi-ExNC

*Withania somnifera* leaf extracts (i-Extract; i-Ex) were prepared as described earlier (1). Ten mg i-Ex, 5 mg DSPE-PEG-Folate (1,2-distearoyl-sn-glycero-3-phosphoethanolamine-N-[folate(polyethylene glycol)]) (Nanosoft Polymers, Lewisville, NC, USA) and 10 mg DSPE-PEG-NH2 (N-(aminopropyl polyoxyethylene oxycarbonyl)-1,2-distearoyl-sn-glycero-3-phosphoethanolamine) (SUNBRIGHT DSPE-020PA; Yuka Sangyo, Tokyo, Japan) were briefly dissolved in 10 ml of double distilled Milli-Q water by bath sonication (power output, 80 W; oscillation frequency, 40 kHz) (Branson 2800; Branson Ultrasonics, Kanagawa, Japan). After gentle stirring for 1 h at room temperature, the mixture was subjected to pulse-type sonication (VCX-600; Sonics, Danbury, CT, USA) for 10 min, followed by centrifugation at 1,000 rpm for 10 min at 20°C (model no. 3740; Kubota, Tokyo, Japan). The FRi-ExNC in the supernatant was collected and used for subsequent experiments. The same protocol was applied for i-ExNC preparation except that the DSPE-PEG-Folate was replaced with an equal mass of DSPE-PEG. Fluorescent nanocomplexes were prepared a the similar way with an additional 1 mg of Nile Red (Wako) in the mixture.

### Characterization of FRi-ExNC

The spectral profiles and concentrations of loaded i-Extract in nanocomplexes were measured with a UV-Vis-NIR spectrophotometer (V-730 BIO; Jasco, Tokyo, Japan). The structure and morphology of prepared FRi-ExNCs were visualized by high-resolution transmission electron microscope (TEM) (EM-002B; Topcon, Tokyo, Japan), an established and suitable method for the investigation of structural aspects of nanomaterials, at an accelerating voltage of 120 kV. Before the TEM experiments, a nanocomplex solution was deposited on a carbon-coated support and negatively stained using a 1% phosphotungstic acid solution. The size distribution was analyzed using Image-J from TEM images (≥200 particles were measured).

### Encapsulation Efficiency of i-Ex

The encapsulation efficiency of i-Ex into FRi-ExNC was calculated using the following equation: encapsulation efficiency = (W_seed_-W_nonencapsulated_)/W_seed_ ×100%, where W_seed_ is the total amount (weight) of i-Ex initially used for FRi-ExNC preparation, and W_nonencapsulated_ represents the amount of i-Ex that was precipitated after centrifugation during the preparation. Briefly, FRi-ExNC was prepared starting with different weight ratios of crude i-Ex powder and DSPE-PEG polymer as indicated. The amount of i-Ex used in this step was recorded as W_seed_. After normal preparation processes, the FRi-ExNCs were subjected to centrifugation at 1,000 rpm for 30 min. The precipitated pellets were then dissolved in 200 μl of dimethyl sulfoxide (Wako) and i-Ex was measured by UV-Vis-NIR spectrophotometer. The amount of i-Ex in pellets (W_nonencapsulated_) was determined based on the absorption at 660 nm by reference to its calibration curve.

### Cell Culture

All cell lines were obtained from the Japanese Collection of Research Bioresources Cell Bank (Tokyo, Japan) or DS Pharma Biomedical (Tokyo, Japan) and cultured in Dulbecco's Modified Eagle's Medium (Gibco, Grand Island, NY, USA) containing 10% fetal bovine serum, 2 mM l-glutamine, 1 mM sodium pyruvate, gentamycin, penicillin-streptomycin (100 IU ml^−1^), and Hank's balanced salt solution (Life Technologies, Carlsbad, CA, USA). Cells were kept in folate-free medium for at least 3 days before and during all the treatments.

### Cytotoxicity Evaluation

Cell viability was assessed with a Cell Counting Kit (CCK)-8 (Dojindo Laboratories, Kumamoto, Japan) following the manufacturer's instructions. Cells (5 ×10^3^ cells well^−1^) were seeded in a 96-well plate and allowed to adhere to the substratum overnight. They were then exposed to i-Ex loaded nanocomplexes as indicated. After washing with a fresh medium, the cells were incubated with CCK-8 solution for 2 h at 37°C. Absorbance at 450/690 nm was read on a microplate reader (Infinite M200 PRO; Tecan, Männedorf, Switzerland). A colony-forming assay was performed to evaluate long-term cytotoxicity of FRi-ExNC. Cells were seeded in a 12-well plate at a density of 200 cells well^−1^. After attachment, they were subjected to treatments for 6 h and allowed to foster in fresh medium for ≥10 days. The forming colonies were washed in cold PBS and fixed with a pre-chilled methanol/acetone (v/v, 1:1) mixture for 10 min. The fixed cells were stained overnight with a 0.1% crystal violet solution (Wako, Osaka, Japan). The colony-forming number was calculated as the mean number of colonies from three independent experiments.

### Immunoblotting

Cells were harvested in radioimmunoprecipitation assay buffer (Thermo Fisher Scientific, Waltham, MA, USA) supplemented with a protease inhibitor cocktail (Roche Applied Science, Mannheim, Germany). The protein concentration was determined using a Pierce BCA Protein Assay kit (Thermo Fisher Scientific). Protein lysate (20 μg) was resolved in SDS-polyacrylamide gels, transferred to PVDF membrane and then probed with antibodies specific to: folate receptor alpha (FR-α) (ab125030; Abcam, Cambridge, MA, USA); Cyclin D1 (72-13G), CDK4 (C-22), and PARP-1 (H-250) (all from Santa Cruz Biotechnology, Santa Cruz, CA, USA); p21^WAF−1^ (12D1) and Bcl-2 (2876S) (both from Cell Signaling Technologies, Danvers, MA, USA); and Caspase-3 (610322; BD Transduction Laboratories, San Diego, CA, USA). The membranes were next incubated with the respective secondary antibodies (Thermo Fisher Scientific). Anti-β-actin (AC-15; Abcam) and anti-β-tubulin (T5293; Sigma-Aldrich, St. Louis, MO, USA) antibodies were used as internal loading controls. The protein bands were quantitated using ImageJ software.

### Fluorescence Microscopy Imaging

Cells (1 ×10^5^ cells well^−1^) were plated on glass coverslips placed in 12-well culture dishes. Nile Red-labeled nanocomplexes were added with or without free folic acid (10 μM) (Wako) when cells had attached to the substratum. After 6 h incubation, cells were washed 2–3 times with cold PBS and fixed in 4% formaldehyde for 10 min. Fixed cells were washed thrice with PBS, followed by nuclear staining with Hoechst 33342 (1 μg ml^−1^; Thermo Fisher Scientific) for 10 min. After serial washings with PBS, coverslips were mounted and visualized under a microscope (Axiovert 200 M; Carl Zeiss, Tokyo, Japan). For immunostaining of FR-α, the fixed cells were permeabilized using 0.5% Triton X-100 in PBS for 10 min and blocked using 2% BSA in PBS for 15 min. Coverslips containing cells were then incubated with anti-FR-α monoclonal antibody (548908; Thermo Scientific) overnight at 4°C, washed thrice with 0.2% Triton X-100 in PBS (PBS-T) and incubated with Alexa Fluor-conjugated secondary antibodies (Thermo Fisher Scientific). After three to six washings with PBS-T, the coverslips were mounted for microscopy imaging.

### Quantification of Cellular Uptake

Quantification of cellular uptake was as described elsewhere (43). Briefly, the calibration curve of i-Ex amount was first established by measuring the absorbance at 660 nm of i-ExNC dispersions at various known concentrations. Cells (2 ×10^5^ well^−1^) were seeded in 6-well plates and incubated overnight to allow attachment followed by treatment with i-ExNC- or FRi-ExNC-supplemented (i-Ex: 20 μg ml^−1^) folate-free medium for 6 h. After several (three to six) washings with PBS, cell pellets were collected and resuspended in RIPA buffer, followed by 30 min sonication. One portion of the cell lysate was used for protein estimation (Pierce BCA Protein Assay kit; Thermo Fisher Scientific). The absorbance at 660 nm of the remaining cell lysates was measured on a microplate reader (TACAN) in triplicate. The concentration of i-Ex in cell lysates (i-Ex/protein) was estimated based on the corresponding calibration curves.

### Flow Cytometry

Cells were seeded at a density of 2 ×10^5^ cells well^−1^ in 6-well plates and harvested by trypsin after treatments as indicated. The cell pellets were fixed with ice-cold 70% ethanol overnight at −20°C. To avoid false DNA-PI staining, RNA was removed by incubation with RNase A (20 μg/mL; Thermo Fisher Scientific) at 37°C for 1 h followed by centrifugation (500 g) for 5 min. _The supernatant was then replaced with 200 μl of Guava Cell Cycle reagent (Millipore, Billerica, MA, USA) and incubated in the dark for 30 min. The stained cells were subjected to cell cycle analysis using Guava PCA flow cytometer (Millipore). Apoptotic cells were detected by Annexin-V and 7-aminoactinomycin (7-AAD) double staining: after treatments, attached and resuspended cells were harvested and apoptosis was analyzed with a Guava PCA flow cytometer (Millipore) using Guava Nexin Reagent (Millipore), a pre-made cocktail containing phycoerythrin-conjugated Annexin V and 7-AAD following the manufacturer's protocol. TdT-mediated dUTP-biotin nick end labeling (TUNEL) assay was performed using a Guava® TUNEL Kit (Millipore) following the manufacturer's instructions. In brief, control and treated cells (1 ×10^5^) were fixed with 4% paraformaldehyde at 4°C for 1 h, and then permeabilized using 70% (v/v) ice-cold ethanol overnight at −20°C. The cells were incubated with TdT reaction mixture containing TdT enzyme and Brd-UTP for 1 h, followed by immunostaining with anti-Brd-UTP antibody (37°C for 30 min). The stained cells were recorded on the Guava system. Positive and negative controls supplied in the kit were used in each assay. The cell cycle distribution and fraction of apoptotic cells were determined using FlowJo v.7.6 software (Tree Star, Ashland, OR, USA).

### *In vivo* Anti-tumor Assay

BALB/c nude mice (4 weeks old, female) were obtained from Charles River Laboratories (Yokohama, Japan). Mice bearing HeLa and HT-29 cell-derived tumors were generated by subcutaneously injecting 1 ×10^6^ cells in 100 μl mixture of culture medium and Matrigel (Dow Corning, Corning, NY, USA) mixture (v/v, 1:1) into the flanks of the mice. Intraperitoneal injections of nanocomplexes containing an equivalent dose of i-Extract (50 mg Kg^−1^) were started when small tumor buds were formed at about 10 days. Saline and free i-Extract (50 mg Kg^−1^) in 10% Cremophor EL (Sigma-Aldrich) were used as the negative and positive controls, respectively. Injections were continued every alternate day, and the mice were monitored for tumor size and overall health (body weight) until 3 weeks. Tumor volume was calculated as V = L × W^2^/2, where L and W are the length and width of the tumor, respectively. All procedures were carried out in accordance with the Animal Experiment and Ethics Committee, Safety and Environment Management Division, National Institute of Advanced Industrial Science & Technology (AIST), Tsukuba, Japan.

### Statistical Analysis

Results are presented as mean ± standard deviation of at least three independent experiments. The numbers of samples per group in each experiment are indicated in the corresponding figure legends as “n.” Differences between groups were evaluated with the Student's *t*-test for two groups and two-way analysis of variance (ANOVA) followed by Tukey test for multiple groups. ^*^, ^**^, and ^***^ denote the *p*-values <0.05, 0.01, and 0.001, respectively.

## Results and Discussion

### Formation and Characterization of FRi-ExNC for Targeted Drug Delivery

**Figure 1:**
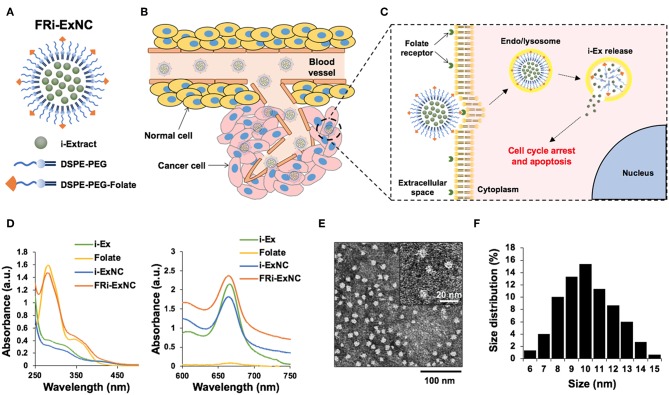
Formation and characteristics of FRi-ExNC for targeted drug delivery. **(A)** An illustration of folate receptor-targeted i-Extract nanocomplex (FRi-ExNC) formed from self-assembly of amphiphilic block copolymers (DSPE-PEG) with folate ligands. **(B)** Sequence of events leading to tumor regression after FRi-ExNC administration: long circulating nanocomplexes were accumulated in the tumor site by EPR effect and **(C)** internalized into cells via folate receptor-mediated endocytosis. The low pH environment of the endo/lysosomes decomposed micelles to release i-Extract into the cytoplasm, resulting in cell cycle arrest and apoptosis. **(D)** UV-Vis-NIR absorption spectra of i-Ex, folate, i-ExNC, and FRi-ExNC showing successful encapsulation of i-Ex in the fabricated micelles. Characteristic peaks of folate at 281 nm (left panel) and i-Extract at 660 nm (right panel) were simultaneously observed in the FRi-ExNC nanocomplex. **(E)** Representative TEM image (a magnified image is shown in the top right corner) and **(F)** quantitative size distribution of FRi-ExNC showing homogeneous shape with size ranging from 6 to 15 nm.

Polymeric micelles modified by FR-PEG-lipid have been shown to be effective for targeted drug delivery to cancer cells (44–46). Thus, we designed the encapsulation of i-EX in FR-PEG-lipid using the polymeric micelle system, examined its characteristics as well as anticancer effects in *in vitro* and *in vivo* assays. The schematic diagram of FRi-ExNC is shown in Figure 1A. The polymeric micelles containing FRi-ExNC can be easily synthesized through a simple self-assembly technique without any complicated multistep processes. i-Ex was encapsulated in the inner core of micelles that were composed of phospholipid-PEG (DSPE-PEG) (for more details, please see Materials and Methods). The folate derivative of DSPE-PEG formed an outer monolayer shell. Such amphiphilic assembly of DSPE-PEG and DSPE-PEG-folate was expected to be capable of encapsulating hydrophobic i-Ex with high loading capacity, and to generate a uniform polymer micelle structure that could target cancer cells selectively (Figures 1B,C). Ultraviolet-visible-near infrared (UV-Vis-NIR) spectroscopy of FRi-ExNC showed a characteristic peak of i-Ex at ~660 nm, confirming the successful encapsulation of i-Ex in the DSPE-PEG and DSPE-PEG-folate micelles (Figure 1D). Of note, the encapsulated i-Ex showed dramatic improvement. As shown in Figure S1, the FRi-ExNC solution was clear and transparent even after storing for 1 week at room temperature; i-Ex solution showed aggregates/precipitates and became turbid (Figure S1). Encapsulation efficiency of i-Ex was increased and reached ~ 87% when i-Ex and DSPE-PEG were used at a 1:3 ratio (Table 1). These data strongly suggested that DSPE-PEG is very useful for solubilizing i-Ex in water. Although scanning electron microscopy (SEM) can provide detailed information about surface structures, it is generally difficult to observe fragile organic samples in high magnification. Furthermore, SEM requires a multistep process for biological samples. On these premises, we opted to do analysis of FRi-ExNC by transmission electron microscopy (TEM). TEM observations revealed that FRi-ExNC contained particles of homogeneous shape and size ranging from 6 to 15 nm (Figures 1E,F). Through repeated experiments, we confirmed that the shape and size of FRi-ExNC particles were constant, stable and highly reproducible. In view of these criteria and ease of preparation of FRi-ExNC, we next investigated its anticancer efficacy in *in vitro* and *in vivo* assays.

**Table 1:** Encapsulation efficiency of FRi-ExNC prepared with different weight ratios of crude i-Extract powder and DSPE-PEG polymers.

**i-Extract/Polymer (w/w)**	**Encapsulation efficiency (%)**
1: 3	87.57 ± 2.31
1: 1.5	66.75 ± 0.57
1: 0.75	49.86 ± 2.08

### Selective Cancer Killing Activity of FRi-ExNC *in vitro*

**Figure 2:**
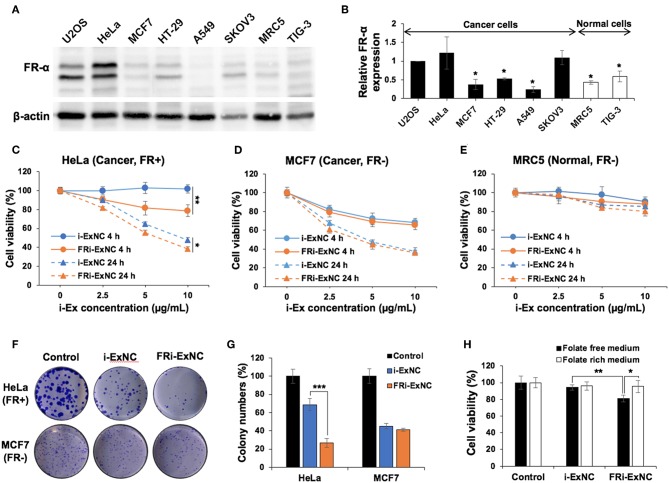
Selective cancer killing activity of FRi-ExNC *in vitro*. **(A)** Western blotting of folate receptor α (FR-α) expression in various cancer and normal cells. **(B)** Quantitation of western blotting for FR-α from three independent experiments is shown on the right (mean ± s.e.m., *n* = 3), ^*^P <0.05 (Student's *t*-test to HeLa). Cell viability assay showing dose- and time-dependent cytotoxicity of FRi-ExNC in **(C)** HeLa and **(D)** MCF7 cancer cells, but not in **(E)** MRC5 normal cells. Folate-conjugated nanocomplexes enhanced selective anticancer activity of i-Ex in FR-α-positive HeLa cells (mean ± s.e.m., *n* = 5), ^*^P <0.05, ^**^P <0.01 (two-way ANOVA test). **(F)** Crystal violet staining in control and nanocomplex-treated cells show that FRi-ExNC caused a stronger reduction of the colony-forming ability in HeLa cells. i-Ex concentration: 5 μg ml^−1^. **(G)** Quantitation from three independent experiments is shown on the right (mean ± s.e.m., *n* = 3), ^***^P <0.001 (Student's *t*-test). **(H)** Cell viability of HeLa cells treated with i-ExNC and FRi-ExNC for 4 h in the culture medium with or without folic acid (mean ± s.e.m., *n* = 5), ^*^P <0.05, ^**^P <0.01 (Student's *t*-test). Stronger cytotoxicity of FRi-ExNC in folate-free medium indicates its favorable tumor-targeting ability toward folic acid receptor-positive cancer cells. i-Ex concentration: 5 μg ml^−1^.

In order to compare the biological activity of i-Ex and FRi-Ex nanocomplexes, we first examined the level of FR-α expression in a variety of cells. As shown in Figures 2A,B, Western blotting with a specific anti-FR-α antibody revealed its enrichment in cancer cells, including U2OS, HeLa, and SKOV3. On the other hand, MCF7, HT-29 and A549 showed expression levels similar to the normal cells (MRC5 and TIG3). Based on the expression data, we selected HeLa (FR-α positive, FR+), and MCF7 (FR-α negative, FR-) cancer cells for further analysis.

*In vitro* cytotoxicity of i-Ex and FRi-Ex nanocomplexes was evaluated with a water-soluble tetrazolium (WST)-based assay after 4 and 24 h treatments. A dose- and time-dependent cytotoxic response to i-ExNC and FRi-ExNC was observed in HeLa (FR+) cells (Figure 2C). Of note, FRi-ExNC showed greater toxicity at all doses as compared to i-ExNC, indicating a favorable folate targeting efficiency. In contrast, MCF7 (FR-) did not show any significant difference in viability in response to either i-ExNC or FRi-ExNC treatment (Figure 2D). Most importantly, the designed FRi-ExNC exhibited only negligible toxicity to normal fibroblasts, MRC5 (Figure 2E). We next performed long term viability (clonogenicity) assays following 6 h of treatment with either i-ExNC or FRi-ExNC. As shown in Figures 2F,G, FRi-ExNC caused a stronger decrease in colony-forming ability in HeLa cells. As folate is required for cell proliferation (47, 48), FRi-ExNC with the folate ligands on the outer surface would theoretically exhibit a higher affinity to the cells undergoing folate starvation. To test this, we incubated HeLa cells with i-ExNC/FRi-ExNC in a culture medium with or without folate for 4 h. As shown in Figure 2H, in the folate-rich medium, neither i-ExNC nor FRi-ExNC showed noticeable toxicity after 4 h of treatment. However, FRi-ExNC cytotoxicity was detected upon a subsequent culture in the folate-free medium; i-ExNC effect remained unchanged (Figure 2H). A conspicuous difference was observed when incubation time was prolonged to 24 h (Figure S2). These results demonstrated that FRi-ExNC could effectively kill FR-α positive cancer cells *in vitro* and was safe to normal cells.

### Selective Cellular Uptake of FRi-ExNC by FR-α Positive Cancer Cells

In order to investigate whether such selective anticancer activity was mediated by folate receptor-mediated selective internalization, a hydrophobic fluorescein molecule (Nile Red) was co-assembled with i-Ex into the nanocomplexes and thus, fluorescent nanocomplexes could be used for bioimaging.

**Figure 3:**
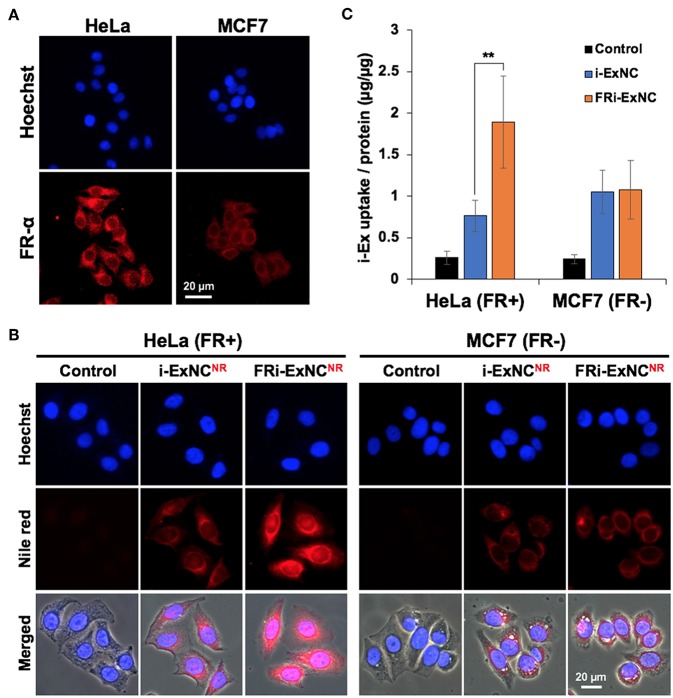
Selective cellular uptake of FRi-ExNC by FR-α-positive cancer cells. **(A)** Immunostaining for FR-α shows cellular membrane and cytoplasm distribution of FR-α, and higher expression in HeLa cells. Nucleus was stained with Hoechst. **(B)** Fluorescence microscopy imaging of HeLa and MCF7 cells incubated with i-ExNC and FRi-ExNC (labeled with Nile Red) for 6 h. FRi-ExNC was more efficiently internalized by HeLa (FR+) cells. **(C)** Quantitative cellular uptake amount of i-ExNC and FRi-ExNC in HeLa and MCF7 cells after 6 h incubation (mean ± s.e.m., *n* = 3), ^**^P <0.01 (Student's *t*-test). FRi-ExNC incubation showed increase in i-Ex uptake by HeLa (FR+) cells, but not MCF7 (FR-) cells.

We first confirmed the expression of FR-α and its localization by immunostaining. As expected, HeLa cells showed higher levels of expression of FR-α, which was mostly expressed on the plasma membrane, compared with MCF7 cells (Figure 3A). We next incubated the cells with Nile Red-labeled i-ExNC (i-ExNC^NR^) and FRi-ExNC (FRi-ExNC^NR^) for 6 h to observe cellular uptake behavior. Fluorescence microscopy imaging revealed that as compared to i-ExNC^NR^, the folate-conjugated composite (FRi-ExNC^NR^) was more efficiently internalized by HeLa (FR+) cells (Figure 3B). Furthermore, we noticed nuclear staining of FRi-ExNC^NR^ that overlapped with Hoechst staining (blue) suggesting that the small size of FRi-ExNC (diameter ≈10 nm) (Figure 1F) is suitable for its internalization into the nucleus. On the other hand, and as expected, MCF7 (FR-) cells did not show any difference in the uptake of nanocomplexes with or without folate (Figure 3B). We also performed an antagonist experiment by co-incubating the cells with an excess of folate (10 μm) and found a decrease in the cellular uptake of FRi-ExNC (Figure S3).

Making use of the unique optical absorption peak of i-Ex (660 nm) at NIR region (Figure 1D), FRi-ExNC could be detected by UV-Vis-NIR spectroscopy with low noise background despite its presence in the cell lysate. We next determined the relative uptake efficiency of i-ExNC and FRi-ExNC by cells; cells treated with either of these compositions were subjected to lysis and spectrophotometric measurement of i-Ex. The quantitative results revealed that i-Ex uptake efficiency was increased ~2.5 times (from 0.76 of i-ExNC to 1.89 of FRi-ExNC) in HeLa (FR+) cells but remained unchanged in MCF7 (FR-) cells (Figure 3C). These data were consistent with fluorescence imaging results and demonstrated that FRi-ExNC could penetrate into cells via folate receptor-mediated endocytosis, which offer enhanced targeting of cancer cells.

### FRi-ExNC Caused Stronger G2/M Arrest and Apoptosis in FR-α-Positive Cancer Cells

**Figure 4:**
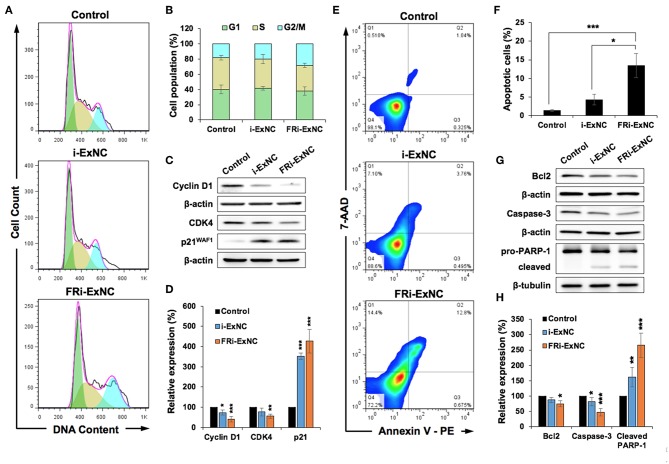
FRi-ExNC caused stronger G2/M arrest and apoptosis in FR-α-positive HeLa cells. **(A)** Cell cycle analysis showing increase in cell population at G2/M arrest by FRi-ExNC treatment. Cells treated with i-ExNC do not show a similar increase at the equivalent dose. **(B)** Quantitation of cell cycle distribution from three independent experiments. **(C)** Western blotting for molecular markers of G2/M transition showing a stronger decrease in the expression of Cyclin D1 and CDK4 in FRi-ExNC-treated cells. p21^WAF1^ was up-regulated after FRi-ExNC treatment. **(D)** Quantitation from three independent experiments is shown below (mean ± s.e.m., *n* = 3), ^*^P <0.05, ^**^P <0.01, ^***^P <0.001 (Student's *t*-test to Control). **(E)** Flow cytometric analysis showing a greater increase in apoptotic cell numbers with FRi-ExNC treatment. **(F)** Quantitation of apoptotic cells from three independent experiments (mean ± s.e.m., *n* = 3), ^*^P <0.05, ^**^P <0.01 (Student's *t*-test). **(G)** Representative blots of apoptotic proteins. Treatment with FRi-ExNC shows a stronger decline in the levels of Bcl-2 and pro-caspase 3. A higher expression level of cleaved PARP-1 was observed in FRi-ExNC-treated cells. Quantitation (mean ± s.e.m., *n* = 3) is shown in **(H)**. ^*^P <0.05, ^**^P <0.01, ^***^P <0.001 (Student's *t*-test to Control). HeLa cells for all the experiments were treated with equivalent dose of i-Ex (5 μg ml^−1^) for 24 h.

In order to obtain molecular insights into the anticancer activity of FRi-ExNC, we performed a cell cycle analysis and found a significant increase in the number of HeLa cells in the G2/M phase after FRi-ExNC treatment (Figure 4A). The growth arrest caused by FRi-ExNC (28% of cells in G2/M phase) was stronger than that caused by i-ExNC (20% of cells in G2/M phase), suggesting that the folate conjugate improved the potency of i-Ex (Figure 4B). The suppression of cell proliferation after nanocomplex treatment was also investigated by Ki-67 immunostaining, an established marker of cell proliferation. As shown in Figure S4A, FRi-ExNC-treated cells showed the strongest decrease in the number of Ki-67-positive cells. Quantitation of the results in the control, i-ExNC and FRi-ExNC groups showed 94.9, 49.0, and 23.6% Ki-67 positivity, respectively (Figure S4B). In order to test this further, we next examined the expression of Cyclin D1, Cyclin-dependent kinase 4 (CDK4), and p21^WAF1^, key regulators of cell cycle progression (49). Western blot analysis showed a stronger decrease in Cyclin D1 and CDK4 in FRi-ExNC- treated, as compared to i-ExNC-treated, cells (Figures 4C,D). Furthermore, the level of p21^WAF1^ was significantly upregulated in response to FRi-ExNC treatment (Figures 4C,D), further confirming the occurrence of growth arrest.

Since i-Ex has been shown to instigate apoptosis at high doses in cancer cells (1), we next examined apoptosis in the control and nanocomplex-treated cells by Annexin V/7-AAD double staining. As shown in Figure 4E, a higher extent of apoptosis was observed in FRi-ExNC-treated, as compared to i-ExNC-treated, cells (Figure 4E). Notably, with the aid of a folate ligand, the proportion of apoptotic cells was elevated from 4.3% (i-ExNC) to 13.5% (FRi-ExNC) (Figure 4F). Similarly, the amount of late apoptotic cells, as examined by TUNEL assay, was 54.4% in the FRi-ExNC-treated cells which was higher than 38.3% for the i-ExNC-treated cells (Figure S4C). Western blotting of proteins critical for apoptosis (50) also revealed that anti-apoptosis factors, B cell lymphoma 2 (Bcl-2), and pro-caspase 3, were downregulated in the FRi-ExNC-treated cells (Figures 4G,H). Immunostaining with a specific anti-cleaved caspase 3 antibody confirmed its upregulation in FRi-ExNC-treated cells (Figure S4D). Consistent with this, cleaved poly-ADP ribose polymerase (PARP)-1 (pro-apoptosis marker) also showed an increase (Figures 4G,H). Altogether, these results demonstrated that FRi-ExNC exerts anticancer activity by inducing cell cycle arrest and apoptosis, and folate conjugates enhanced its potency in FR-α-positive cancer cells.

### *In vivo* Anticancer Effect of FRi-ExNC on Enhancing Selective Targeting of FR-α-expressing Tumors

**Figure 5:**
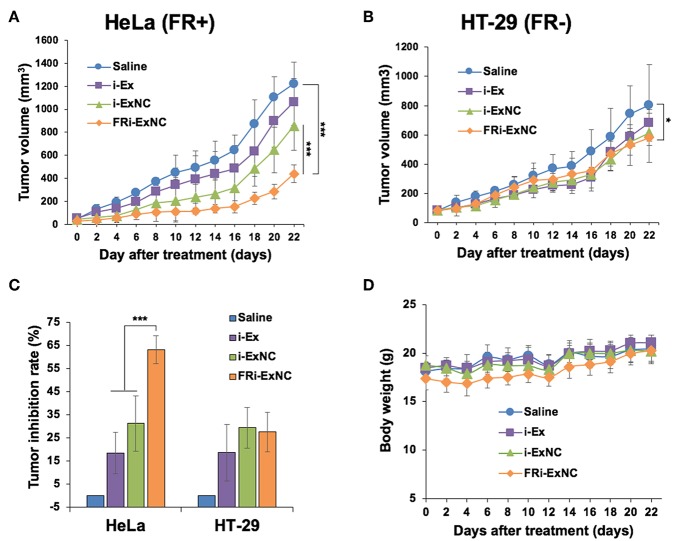
*In vivo* anticancer effect of FRi-ExNC on enhancing selective targeting of FR-α-expressing tumors. Relative HeLa **(A)** and HT-29 **(B)** tumor volumes in different groups of mice during the course of treatment (mean ± s.e.m., *n* = 5), ^***^P <0.001 (two-way ANOVA test). Subcutaneous xenografts of HeLa (FR+) cells in nude mice treated with FRi-ExNC showed low tumor-forming capacity as compared to HT-29 (FR-) cells. **(C)** Inhibition rate of HeLa and HT-29 xenografts in nude mice with different treatments at the end of the experiment (mean ± s.e.m., *n* = 5), ^*^P <0.05 (Student's *t*-test). The data indicates that FRi-ExNC has the selectivity to impede HeLa (FR+) tumor progression. **(D)** The average body weight of mice did not show any loss during treatments. All the treatments were performed with an equivalent dose of i-Ex (50 mg/Kg/2 days).

We next performed *in vivo* assays to evaluate the anti-tumor effect of FRi-ExNC using a subcutaneous xenograft nude mouse model. As expected, MCF7 cells did not yield solid tumors. On the other hand, HT-29 cells formed tumors with a growth similar to HeLa cells. Due to these technical reasons and to compare the effect of FRi-ExNC on tumors with a similar growth rate, HT-29 and HeLa cells were used *in vivo*. HeLa (FR+) and HT-29 (FR-) tumor-bearing nude mice with an initial tumor volume of ~100 mm^3^ were randomly assigned to one of four groups that were administered an equivalent dose (50 mg Kg^−1^ Body Weight) of i-Ex alone or encapsulated in nanocomplexes (i-ExNC and FRi-ExNC) through intraperitoneal injections. Saline was used as a blank control. For both cell lines, tumors showed progressive growth and reached 1.1–1.4 cm (diameter) and a tumor volume of ~1,200 mm^3^ in about 22 days. i-Ex, i-ExNC, and FRi-ExNC treated mice showed delayed tumor growth as compared to the control group (Figure 5A). Furthermore, HeLa (FR+) tumors showed strong suppression in the FRi-ExNC treatment group as compared to the saline, i-Ex, and i-ExNC groups (Figure 5A). This could be attributed to selective targeting of HeLa (FR+) cells by FRi-ExNC. On the other hand, HT-29 (FR-) tumors also showed growth suppression in the FRi-ExNC group as compared to the saline control. However, the effect was comparable to i-Ex- and i-ExNC-treated groups (Figure 5B). The tumor inhibition rate in response to FRi-ExNC treatment reached 63.1% in the HeLa xenografts, which was much higher than those treated with i-Ex (18.4%) and i-ExNC (31.2%), whereas there was no significant difference between i-ExNC and FRi-ExNC treatments in the HT-29 xenografts (Figure 5C). These results clearly indicated that FRi-ExNC has the selectivity to impede FR-α-positive tumor progression *in vivo*. The significantly enhanced therapeutic efficacy of FRi-ExNC was highly consistent with the *in vitro* observations. In addition, there was no significant loss of body weight in the mice among all treatments (Figure 5D), demonstrating that FRi-ExNC has low systemic toxicity.

In summary, we have prepared a folate receptor-targeting nanocomplex loaded with i-Extract for enhancing its selective toxicity to cancer cells. The functionalized FRi-ExNC spontaneously assembled under physiological condition by simple sonication and was easily resuspended in water with sizes of ~10 nm. With the aid of folate ligands, FRi-ExNC showed a 3-fold higher cellular uptake and induced stronger cytotoxicity to FR-α-enriched cancer cells *in vitro*. *In vivo* anti-tumor assays showed that FRi-ExNC caused stronger tumor suppression of FR-α-positive xenografts. We propose FRi-ExNC as a suitable nanomedicine and platform to facilitate the use of *Withania somnifera* bioactives for cancer treatment and hence warrant further studies and clinical trials.

## Data Availability

The raw data supporting the conclusions of this manuscript will be made available by the authors, without undue reservation, to any qualified researcher.

## Ethics Statement

All animal experiments were performed in strict accordance with protocols approved by the Institutional Animal Care and Use Committee of AIST.

## Author Contributions

RW and EM conceived and designed the experiments. YY and JW performed the experiments and collected and analyzed the data. YY, RW, SK, and EM prepared the manuscript. All authors discussed the results and contributed in writing the manuscript.

### Conflict of Interest Statement

The authors declare that the research was conducted in the absence of any commercial or financial relationships that could be construed as a potential conflict of interest.
