# Supplementary material for: Folic Acid Receptor-Mediated Targeting Enhances the Cytotoxicity, Efficacy, and Selectivity of Withania somnifera Leaf Extract: In vitro and in vivo Evidence
Source: Front Oncol. 2019 Jul 4;9:602. doi: 10.3389/fonc.2019.00602 (PMC6621239; doi:10.3389/fonc.2019.00602)
Supplement: Supplementary file 1 [file Data_Sheet_1.docx]

**Supplementary Material**

**
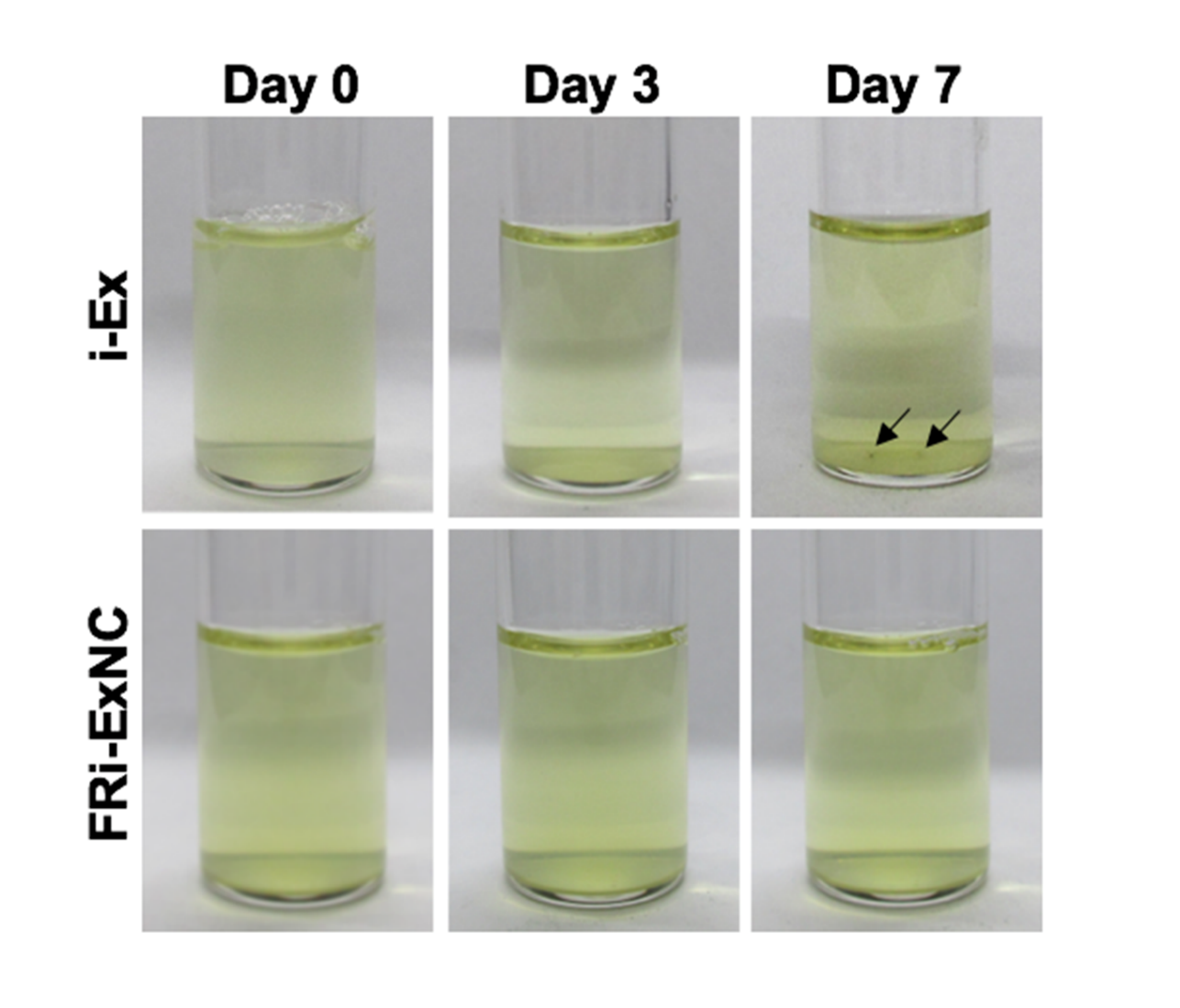
**

**Figure S1** Images of i-Ex and FRi-ExNC water solutions after storing at room temperature for different time periods. The suspension of FRi-ExNC (iEx: 0.5 mg/ml) remained clear until day 7, but i-Ex alone aggregated, became turbid and precipitated, as shown by arrows.

**
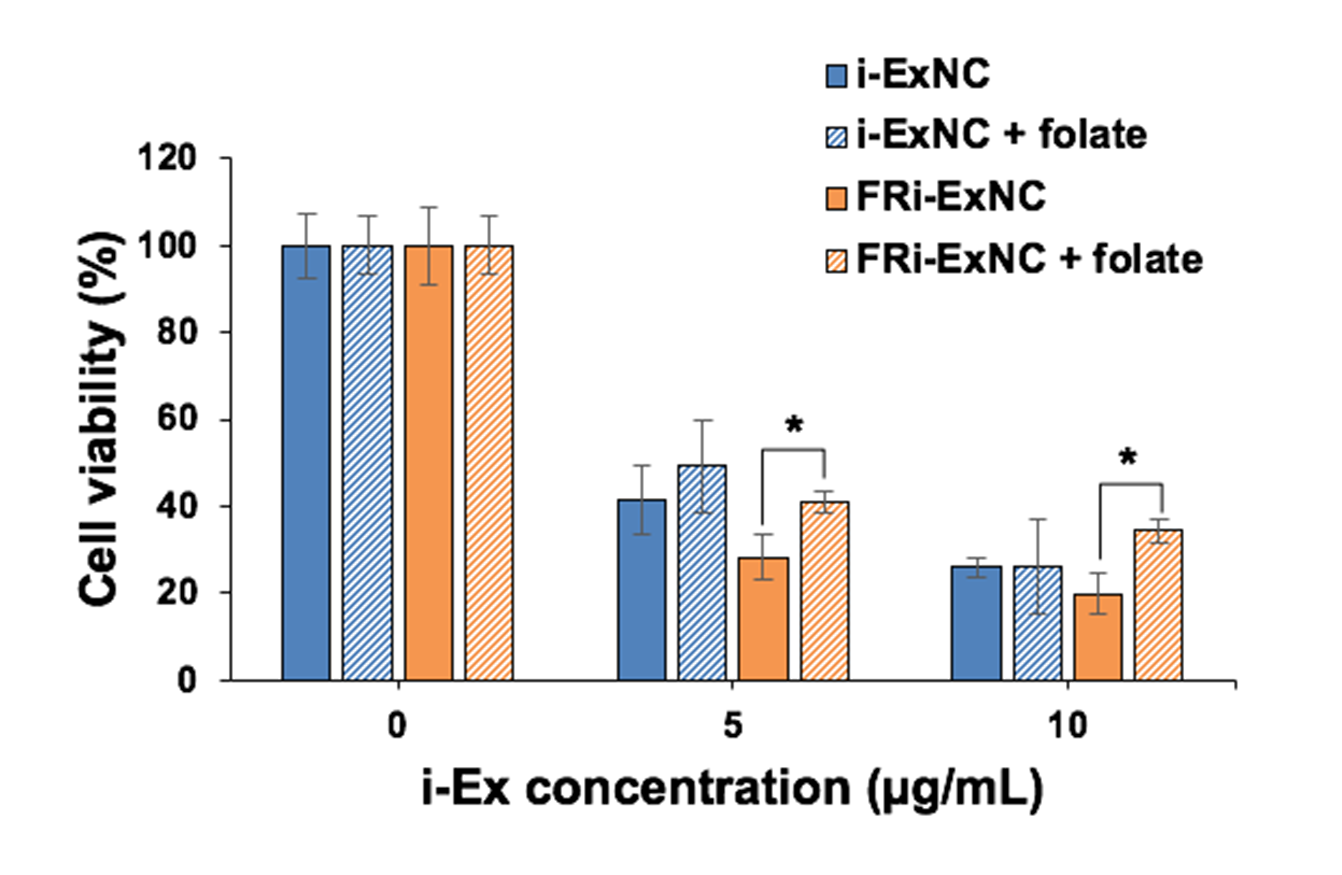
**

**Figure S2** Viability of HeLa cells treated with i-ExNC and FRi-ExNC for 24 h with/without 10 µm free folate (mean ± s.e.m., n = 5), *P<0.05 (Student’s t-test). Stronger cytotoxicity of FRi-ExNC without the addition of folate indicates its favorable tumor-targeting ability towards folic acid receptor-expressing cancer cells.


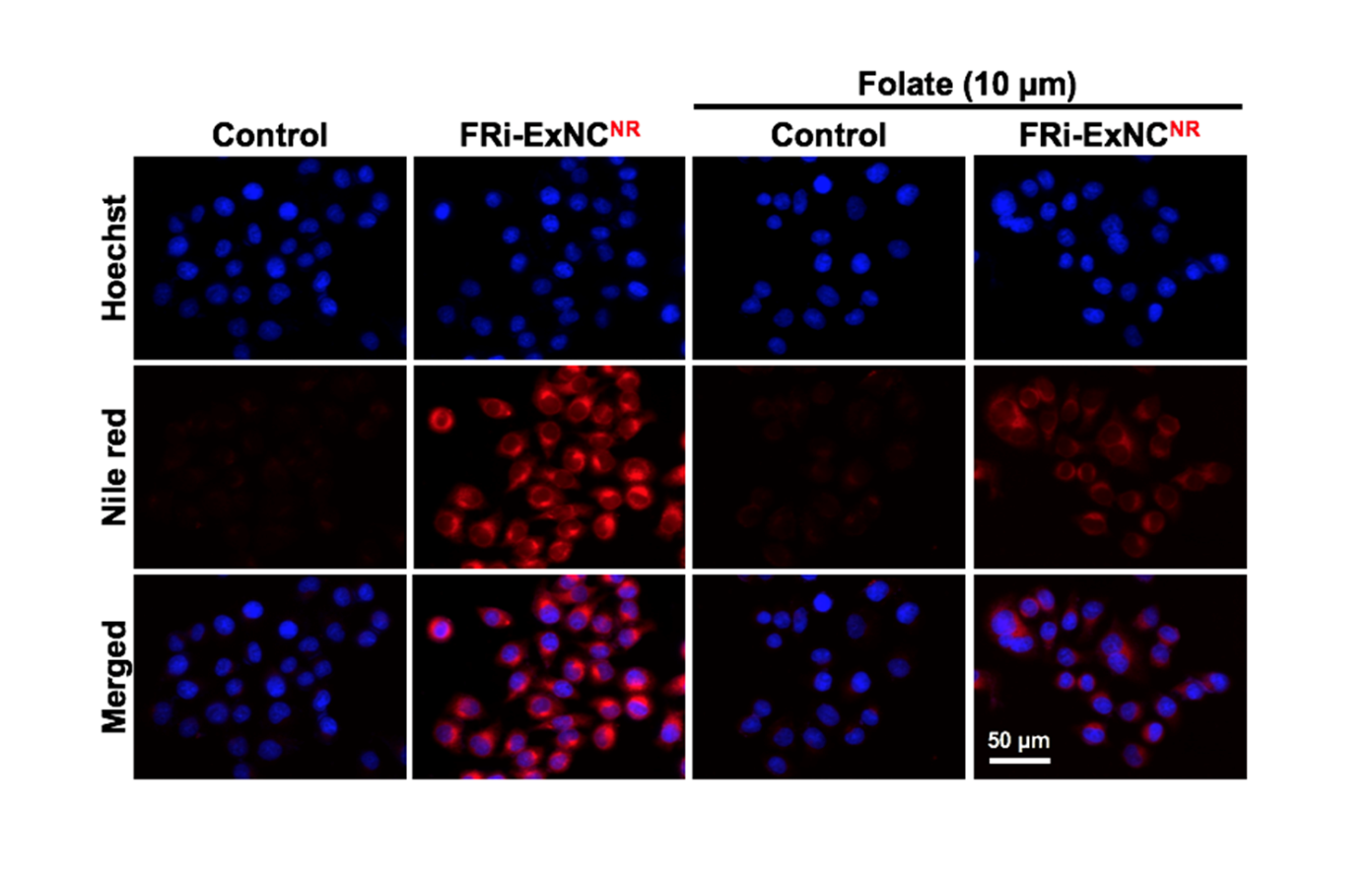


**Figure S3** Fluorescence microscopy imaging of HeLa cells treated with FRi-ExNC (labeled with Nile Red) for 6 h. The internalization of FRi-ExNC was inhibited by co-incubation with a free folate, indicating the successful modification of FRi-ExNC and the folic acid receptor-targeting effect. The nucleus was stained with Hoechst.


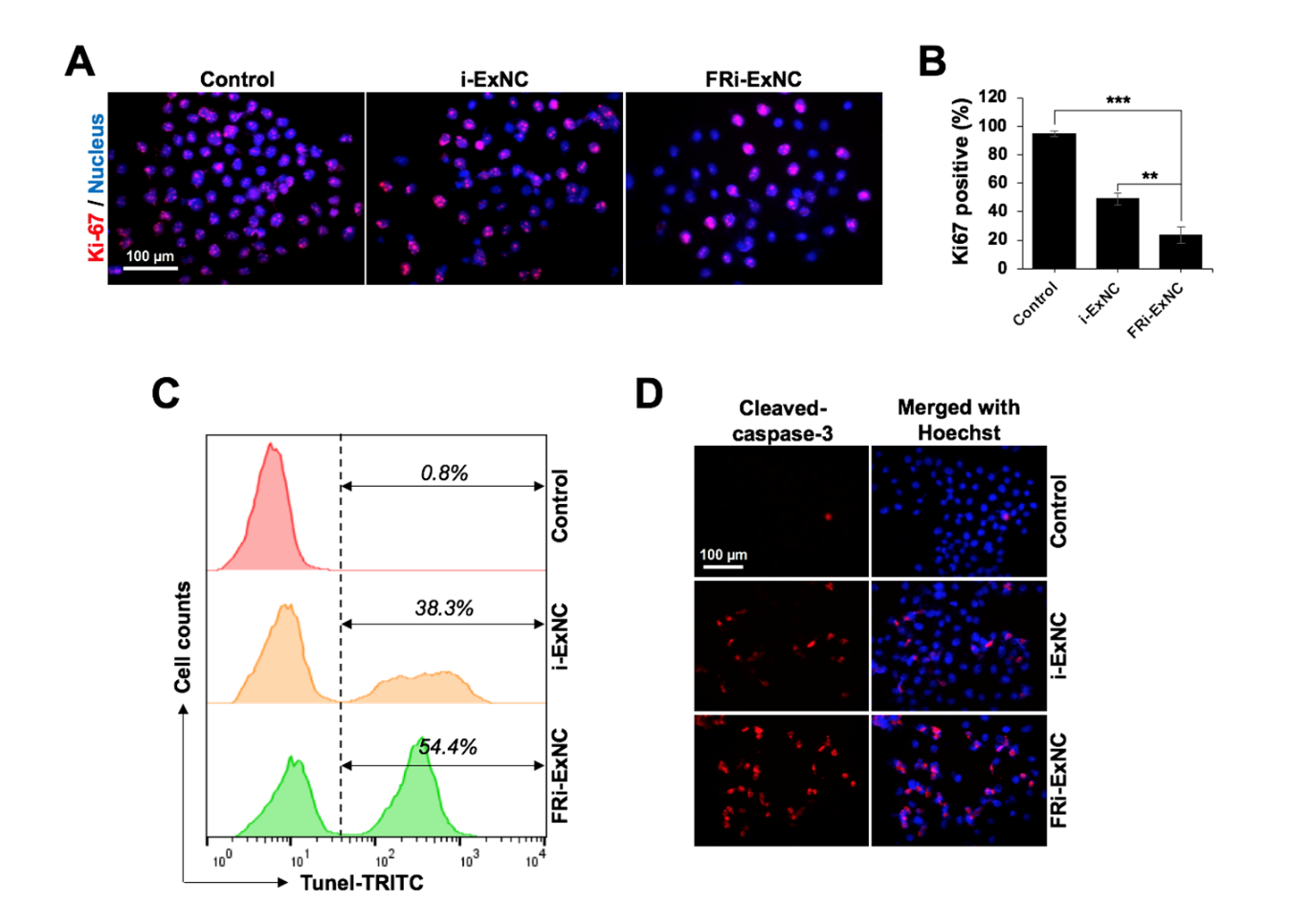


**Figure S4** (A) Immunostaining of HeLa cells showing a decrease in the number of Ki-67-positive cells following i-ExNC and FRi-ExNC treatments. The nucleus was stained with Hoechst. (B) Quantitation from at least 200 cells is shown on the right (mean ± s.e.m., n = 3), **P<0.01, ***P<0.001 (Student’s t-test). FRi-ExNC group showing the largest reduction. (C) TUNEL assay and (D) immunostaining for cleaved-caspase 3 in control and nanocomplex-treated HeLa cells show that FRi-ExNC induced a greater apoptosis than i-ExNC. Cells were treated with an equivalent dose of i-Ex (10 µg ml^−1^) for 24 h.
